# The 16^th^ Congress of the Latin Society of Cardiology and Pediatric Cardiovascular Surgery, held in Brazil: an inside out analysis

**DOI:** 10.21470/1678-9741-2018-0603

**Published:** 2018

**Authors:** Walter V. A. Vicente

**Affiliations:** 1 Faculdade de Medicina de Ribeirão Preto da Universidade de São Paulo (FMRP-USP), Ribeirão Preto, SP, Brazil.

The 16^th^ Congress of the Latin Society of Cardiology and Pediatric Cardiovascular Surgery (Sociedad Latina de Cardiología y Cirugía Cardiovascular Pediátrica - SLCCCP) led a 5-day joint meeting held in Brazil, from October 24 to 28, 2017, enrolling 56 international guests from 14 countries, and 67 colleagues from 15 Brazilian states.

The event celebrated the 15^th^ Annual Pediatric Cardiology International Meeting of Ribeirão Preto, in a university hub located 330 km Northeast of São Paulo. It was attended by 116 people from nine countries and granted promotional spaces for several non-governmental foundations dedicated to support children with heart disease in Brazil.

The three-day SLCCCP Congress was preceded by the 5th International Pediatric Cardiology Research Symposium that provided a unique forum for original basic and clinical studies presentations by junior and senior investigators from international and Brazilian centers. Among the eight junior presentations, one on lower extremity lymphatic vessel dynamics in patients with total cavo-pulmonary connection, presented by a medical student from Denmark, was selected as the best paper and the author received an award during the Congress. The international senior speakers came from Aarhus, Denmark; Bonn, Germany; Buenos Aires, Argentina and Boston, USA, whereas three colleagues from São Paulo completed the program.

On the morning of the last day, the International Symposium on Extracorporeal Circulation in Pediatric Cardiovascular Surgery, co-organized by Dr. Luiz Fernando Caneo, from InCor São Paulo, wrapped up scientific activities. It covered recent advances in cerebral neuroprotection, anesthesia in cardiac surgery, safety protocols for extracorporeal circulation, and operating room teamwork. In addition to three national experts, Perfusion Clinical Coordinator at Boston Children's Hospital, Greg Matte, and Prof. Rodolfo Neirotti were the leading invited international speakers.

The Congress' comprehensive scientific program included round tables on transposition of the great arteries (TGA), Ross and Ross-Konno operations, tetralogy of Fallot, Ebstein's malformation, congenital heart disease (GUCH), congenitally corrected transposition of the great arteries (CCTGA), Fontan-Kreutzer operation, mechanical circulatory support, and interventional cardiology. The inclusion of international and Brazilian speakers as well as two commentators from different countries in each round table was seminal to encourage discussion and enhance the audience interest.

In addition, conferences, mini-conferences, special sessions, and oral and poster presentations provided a diversity of captivating topics. Among them, the evidence-based demonstration that pediatric cardiac surgery humanitarian programs contribute to the human development index in emerging countries merits mention, an update of the World Society of Cardiovascular Pediatric Surgery Database, a potpourri cardiac specimens demonstration, evaluation of heart transplantation experiences in Argentina and Brazil, advances in imaging technology, respiratory syncytial virus prophylaxis, stem cell therapy, operability limits in patients with congenital heart disease and pulmonary hypertension and intraoperative myocardial protection in the neonate. Other themes worth mentioning were an innovative investigation of the postoperative univentricular heart circulation lymphatic physiopathology, with infrared fluorescence, results with a patient cohort submitted to lymphatic decompression surgery, "How to do it" surgical videos, an update on percutaneous arrhythmia ablation, and the pros and cons of performing adult and pediatric cardiac surgery under the same roof.

The Society paid a much-deserved tribute to Guillermo Kreutzer, for his contribution to the treatment of the univentricular heart, and invited Miguel Barbero-Marcial to present his lifelong accomplishments and reflections on the specialty.

In the Society's Assembly many colleagues recognized the high standards of the scientific program, the Congress venue and the meeting organization. Furthermore, we were honored with the presidency of the SLCCCP during the 2017-2019 biennium, until the next meeting in Buenos Aires.

After a special word of appreciation to the local nonprofit Sinhá Junqueira Foundation that backed most of the meeting's budget, and before the farewell words by the president, took place a Posthumous Homage delivered by Edmar Atik, from Brazil, to Pedro Antonio Sánchez, a cardiologist from Spain and one of the ancestors of the SLCCCP.

The success of the meeting sends a valuable and persuasive message to the officials in charge of the Brazil's public health system. Considering that the vast majority of pediatric cardiology and pediatric cardiovascular surgery centers were established in state capitals, mostly of them located in the coastal area, the up and coming centers, like ours, that more recently start practicing in the country's interior, must be properly recognized and better nourished. Indeed, these new units deliver high complexity care for the local and regional population, and increase the capacity of the Brazilian national health system to assist patients from remote regions of the country.

Ribeirão Preto, with the second largest affiliated tertiary hospital of the University of São Paulo, is one such example. Its pediatric cardiology group members have obtained postgraduate training in leading institutions abroad and are highly committed to improving clinical results and academic achievements. All considered it is wise and timely to start granting our centers and other similar centers the attention and longed for the funding they need to expand and stand out.

Perseverance and experience accumulated over 14 years of putting together an annual international meeting, a strong and official financial back up from a local private institution, contributions from the University Hospital Foundation for Assistance and Research (Fundação de Apoio ao Ensino, Pesquisa e Assistência do Hospital das Clínicas da Faculdade de Medicina de Ribeirão Preto da Universidade de São Paulo - FAEPA), medical industry and private organizations, as well as the seminal encouragement of the SLCCCP past presidents were our strongholds.

Behind the scenes, in an era of resource scarcity, we must praise the spirit of friendship and cohesion from all the prominent international colleagues who promptly accepted to come to Brazil without any financial help from the Congress, the same holding true for the Brazilian colleagues. The cooperation and suggestions of Prof. Rodolfo Neirotti, a native Spanish-speaking international leader of the specialty, and the Danish group headed by Prof. Vibeke Hjortdal, the support of the Department of Congenital Heart Disease and Pediatric Cardiology of the Sociedade Brasileira de Cardiologia, and the Department of Pediatric Cardiovascular Surgery of the Sociedade Brasileira de Cirurgia Cardiovascular were also primordial.

An in all, we must acknowledge the SLCCCP's widespread recognition by other important peer organizations and by its many Latin languages speaking teams, most of them suffering the tribulations of being part of the underdeveloped world. By avoiding favoritism and inequality, it became a cherished bastion to the advancement of knowledge and highly praised and beloved by its fellow members whose generosity, truly unselfish behavior, exemplary solidarity and an ever-increasing friendship allowed us to put together such a great event.

The author wishes to thank Prof. Rodolfo Neirotti for revising the manuscript and for his thoughtful comments.

